# Cost effectiveness of typhoid vaccination in India

**DOI:** 10.1016/j.vaccine.2021.06.003

**Published:** 2021-07-05

**Authors:** Akashdeep Singh Chauhan, Isha Kapoor, Saroj Kumar Rana, Dilesh Kumar, Madhu Gupta, Jacob John, Gagandeep Kang, Shankar Prinja

**Affiliations:** a Department of Community Medicine and School of Public Health, Postgraduate Institute of Medical Education and Research, Chandigarh 160012, India; b The Wellcome Trust Research Laboratory, Division of Gastrointestinal Sciences, Christian Medical College, Vellore 632 004, India; c Department of Community Health, Christian Medical College, Vellore 632 002, India

**Keywords:** Typhoid, Typhoid conjugate vaccine, Cost effectiveness analysis, India

## Abstract

**Introduction:**

World Health Organization has prequalified the use of typhoid conjugate vaccine (TCV) in children over six months of age in typhoid endemic countries. We assessed the cost-effectiveness of introducing TCV separately for urban and rural areas of India.

**Methods:**

A decision analytic model was developed, using a societal perspective, to compare long-term costs and outcomes (3% discount rate) in a new-born cohort of 100,000 children immunized with or without TCV. Three vaccination scenarios were modelled, assuming the protective efficacy of TCV to last for 5, 10 and 15 years following immunization. Incidence of typhoid infection estimated under ‘National Surveillance System for Enteric Fever’ (NSSEFI)’ was used. The prices of vaccine and cost of service delivery were included for vaccination arm. Both health system cost and out-of-pocket expenditures for treatment of typhoid illness and its complications was included.

**Results:**

TCV introduction in urban areas would result in prevention of 17% to 36% typhoid cases and deaths. With exclusion of indirect costs, the incremental cost per QALY gained was ₹ 151,346 (54,730–307,975), ₹ 61,710 (−5250 to 163,283) and ₹ 45,188 (−17,069 to 141,093) for scenario 1, 2 and 3 respectively. While, with inclusion of indirect costs, all 3 scenarios were cost saving. Further, in rural areas, TCV is estimated to reduce the typhoid cases and deaths by 19% to 36%, with ICER (incremental cost per QALY gained) ranging from ₹ 2340 (1316–4370) to ₹ 3574 (2057 – 6691) thousand (inclusive of indirect costs) among the 3 vaccination scenarios.

**Conclusion:**

From a societal perspective, introduction of TCV is a cost saving strategy in urban India. Further, due to low incidence of typhoid infection, introduction of TCV is not cost-effective in rural settings of India.

## 1 Background

As per the most recent estimates, typhoid fever is a significant public health concern with 10.9 million cases and 117 thousand deaths globally [1]. Although there has been a global decline of 55% in the incident cases and 41% in deaths caused by typhoid over the last 3 decades, yet it remains a major cause of disability and death in low income countries [1]. The South Asia region contributes to around 70% of the global cases as well as mortality due to typhoid fever and India alone accounts for 82% and 75% of this incidence and mortality in South Asia [1].

The transmission of typhoid is mainly through consumption of contaminated food or water, therefore access to safe water, adequate sanitation and appropriate hygiene are central to its prevention. However, the most vulnerable populations in low income countries have poor access to safe water and improved sanitation, which underlines the need of integrating vaccination in the disease prevention and control strategies [2]. The World Health Organization (WHO) has licensed 3 types of typhoid vaccines – typhoid conjugate vaccine (TCV), unconjugated Vi polysaccharide (ViPS) and live attenuated Ty21a vaccine [3]. Although the latter two i.e., ViPS and Ty21a were approved for high endemic countries by WHO in 2000, there has been limited uptake of these vaccines [4]. This is primarily due to the low efficacy, requirement for multiple does and restrictive age limit for those below 2 years [3], [4]. However, the recently developed Typbar-TCV with a higher efficacy (>80%), longer duration of protection (more than 5 years), fewer doses and suitability for children below 2 years of age, makes it a better candidate for inclusion in routine vaccination schedule of children [5], [6], [7], [8], [9]. Typbar-TCV has been approved by WHO and also been recommended for routine use in children over six months of age in typhoid endemic countries by the Strategic Advisory Group of Experts on immunization [7], [9], [10]. Further, to facilitate TCV introduction in developing countries, Global Alliance for Vaccines and Immunization (GAVI) has approved US$ 85 million funding window for the nationwide introduction of TCV in routine immunisation and for catch-up immunisation (up to 15 years of age) as appropriate to a country’s epidemiologic context [11]. This funding is available for covering the costs of vaccine, injection supplies and the introduction costs [11]. As of 2020, 57 countries (including India) across the globe are eligible to apply for new vaccine support from GAVI for the year 2020 [12].

Cost effectiveness analyses (CEA) are a significant tool to guide policymakers in formulating evidence based decisions on introduction and implementation of a new health intervention or program [13]. The National Technical Advisory Group of Immunization in India (NTAGI) has constituted a working group on CEA [14]. Secondly, the Health Technology Assessment in India (HTAIn) also places importance on evidence on cost-effectiveness for allocating scarce health resources to maximize health outcomes [15]. There have been few economic evaluations of typhoid vaccination from India, and these studies have found the vaccination to be cost effective or a cost saving strategy [16], [17], [18], [19]. Among these, two studies were undertaken considering ViPS for individuals above 2 years of age [16], [17]. The studies which evaluated TCV, used the data on incidence and treatment cost which were more than a decade old. Moreover the vaccine delivery cost was normatively derived, rather than based on empirical observation [18], [19]. Further, none of these took into consideration the treatment seeking pattern of typhoid infected patients that might have led to improper assessment of both cost and health outcomes.

In 2017, National Surveillance System for Enteric Fever in India (NSSEFI) was initiated at multiple sites with an aim to estimate a nationally representative data, segregated by urban and rural areas, on the incidence of typhoid infection [20]. It incorporates 3 tiers of surveillance – active community-based, passive hospital-based and laboratory-based surveillance. The data generated from this surveillance on healthcare utilization, out-of-pocket expenditure, quality of life will aid in developing appropriate immunization strategies. Keeping in view the limitations of previous studies on CEA of typhoid vaccines, and the newly available evidence from NSSEFI, the present study was undertaken to assess the cost-effectiveness of TCV in India. Finally, based on the observed urban–rural differences in the incidence of typhoid infection, healthcare utilization patterns, vaccine coverage rates and cost of illness, we assessed the cost-effectiveness of TCV separately for urban and rural areas of India.

## 2 Methods

### 2.1 Overview

**Fig. 1:**
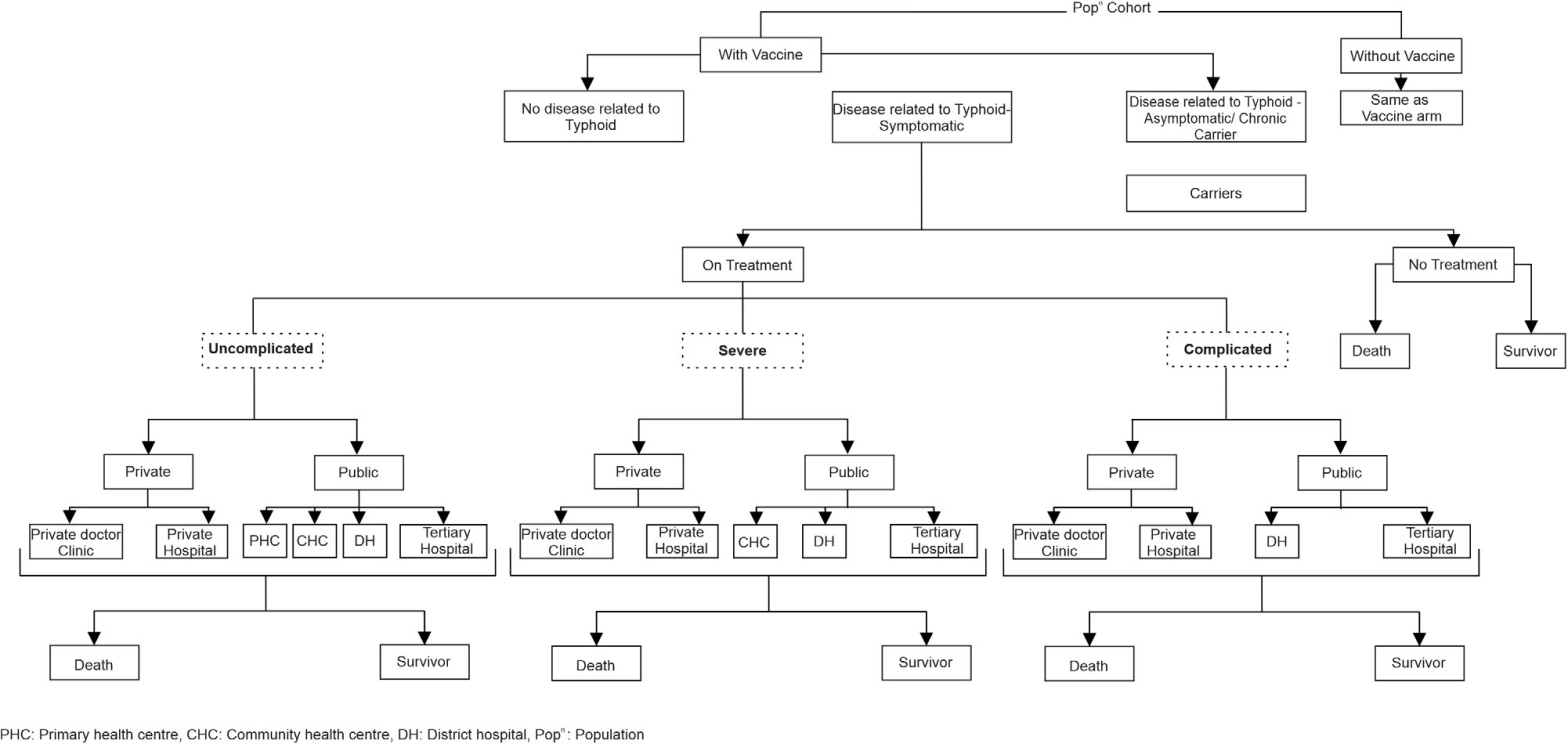
Model structure.

A decision analytic model (Fig. 1) was developed in MS-Excel for comparing both the costs and health outcomes in a new-born cohort of 100,000 children immunized with or without TCV for urban and rural areas of India. The children in the vaccinated arm were assumed to be immunized with TCV at the age of 6 months as per recommended guidelines [9]. The time horizon of the model was taken to be 15 years, based on the duration of the efficacy of TCV and high incidence of typhoid till 15 years of age. The health outcomes were assessed in the form of reduction in mortality, gain in life years (LY) and quality adjusted life years (QALY) with TCV as compared to no vaccination. We used a societal perspective that included health system costs, patient level out-of-pocket expenditures, and indirect costs including productivity losses. A discount rate of 3% was used to discount future cost and consequences [13], [21]. The cycle length of the model was assumed to be monthly, considering the fact that the average duration of typhoid illness is around 3–4 weeks [22]. The cost effectiveness was measured as the ratio of additional (or incremental) costs to additional health benefits i.e., incremental cost effectiveness ratio, with TCV vaccination as compared to no vaccination in India. Based on the guidelines of Health Technology Assessment Board of India, an intervention (i.e., typhoid vaccination) is considered to be cost effective if its incremental cost effectiveness ratio (ICER) falls below the one time GDP per capita of the country [21]. Following the same approach we have also used the GDP per capita value of ₹ 147,854 for the year 2019 as the threshold of cost effectiveness for India.

### 2.2 Model structure

The model structure, as shown in Fig. 1, was developed and used separately as well as independently for assessing the cost effectiveness in urban and rural population. The model starts with the cohort of new-born children assumed to have a risk of developing typhoid infection based on its incidence (separately for urban and rural settings) with or without vaccination (Fig. 1). Infected children, based on the natural history of typhoid and clinical severity of the disease, were categorized into 3 main symptomatic stages i.e., uncomplicated, severe and complicated infection [23], [24]. In the uncomplicated stage, children have a gradual onset of symptoms with fever, headache, malaise, anorexia, lethargy, abdominal pain, diarrhoea, constipation, rose spots etc. [25]. The children with these manifestations are usually treated on an outpatient basis [22]. Further, in some circumstances when the condition of the patient deteriorates due to worsening of the above mentioned symptoms, patient may require hospitalization in the severe stage of the disease. Lastly, typhoid patients may end up having serious complications in the form of gastro-intestinal bleeding or even perforation (specially ileal perforation), hepatitis, pneumonia, urinary tract infection, myocarditis, shock, meningitis, etc. [26], [27]. Patients with complications require intensive inpatient care with medical or surgical management.

It was assumed that the symptomatic children either seek treatment or not, depending on the care seeking behaviour. Those who took the treatment, were assumed to have utilized either of the public (at different levels) or private (both for profit and charitable) health care facility. We did not consider any typhoid specific mortality in the uncomplicated and severe stage of the disease, as patients in these stages were assumed to recover from the illness (based on the therapeutic management as per standard treatment guidelines (STG) of India) [23], [28], [29]. The patients with complications either recover or die, based on the case fatality rate (CFR) for respective complications [27]. Likewise, those who did not undertake any treatment had a probability of dying based on the CFR among those without treatment [30]. Besides the risk of mortality due to typhoid, we also assumed the risk of age specific all-cause mortality for patients in both the treated and untreated arms. (eAppendix: Supplementary material - Table S1) [31].

### 2.3 Illness burden and care seeking behaviour

Incidence of typhoid infection stratified by urban and rural area, and by age (6 months to 5 years; 5–9 years and 10–15 years), as estimated in the community based cohorts by the NSSEFI study across 4 sites in Delhi, Kolkata, Vellore and Pune was used (Table 1) [20]. The proportion of symptomatic patients who sought treatment on an outpatient basis (uncomplicated stage) and those requiring hospitalization (severe or complicated) was also assessed from the NSSEFI study [20]. Among the total hospitalizations, data on segregation of patients into severe cases (i.e., without complications) and those with complications was derived from the retrospective, cross sectional study undertaken in 5 multispecialty hospitals across India [27]. Further, data from this study was also used to assess the proportion of various complications as shown in Table 1. CFR in the complicated stage of the disease was considered to be 6.5% and the mortality rate among those without treatment was assumed to be 20% [27], [30]. Age specific all-cause mortality rates were assessed from Census of India, Sample Registration System life tables [31].

**Table 1:** Model parameters.

**Model parameters**		**Value**	**Range**	**Source**
Incidence of typhoid infection per 100,000 child years in urban settings	6 months to 5 years	713	558–911	[20]
	5–9 years	983	831–1162	
	10–14 years	752	615–918	
Incidence of typhoid infection per 100,000 child years in urban settings	6 months to 14 years	35	3–220	
Proportion of patients in different severity levels	Severe	0.13	±20%	[20], [27], [32]
	Complicated	0.3		
	Without treatment: Urban settings	0.003		
	Without treatment: Rural settings	0.013		
Proportion of patients in various complications	Illeal perforation	0.045	±20%	[26], [27]
	Hepatitis	0.35		
	Encephalopathy	0.16		
	Gastro-intestinal bleeding	0.13		
	Renal impairment	0.10		
	Hemodynamic shock	0.10		
	Myocarditis	0.03		
	Pneumonia	0.045		
	Urinary tract infections	0.015		
	Osteomyelitis	0.015		
Case fatality rate	Complicated	0.065	±20%	[27]
	Without treatment	0.2		[30]
QoL weights	Uncomplicated	0.94	0.89–0.99	a
	Severe	0.89	0.86–0.92	
	Complicated	0.46	0.29–0.64	
	Without treatment	0.94	0.89–0.99	
Coverage of vaccine	Urban settings	64%	±20%	[33]
	Rural settings	61%		
Efficacy of vaccine		87%	±10%	[6], [7], [10]
Herd Immunity		44%	2%−69%	[34]
Cost of vaccination (₹)	Price of vaccine	108	±40%	[10]
	Service delivery cost	144		[35]
Average number of outpatient visits for management of uncomplicated typhoid infection		3	2–5	[20], [27]
Mean length of stay for inpatient care in typhoid infection without complications		6	4–8	
Mean length of stay for inpatient care in typhoid infection with complications		7	5–12	

a: estimates based on primary data collection.

Information on the treatment seeking behaviour for those typhoid infected children ≤ 15 years of age was obtained from National Sample Survey (NSS) – 75th report [32]. Based on this report, data on the utilization rate of public and private facilities, assessed separately for urban and rural areas, for an outpatient visit and hospitalization for the management of all fever cases, in the age group of 0–15 years, were used as proxy for assessing the treatment seeking pattern of typhoid infected children (eAppendix: Supplementary material - Tables S2). Similarly, the prevalence of unmet need (0.3% and 0.13% in urban and rural areas) for all type of fever cases (from NSS – 75th round), was considered for estimating the proportion of those typhoid infected symptomatic patients that did not undertake any treatment.

### 2.4 Vaccination scenarios

Three vaccination scenarios were modelled based on the duration of efficacy of TCV. The first scenario assessed the cost and consequences of vaccinating children with TCV at 6 months, and assumed the protective efficacy to last for 5 years. Similarly, the 2nd and 3rd vaccination scenarios assessed the effect of TCV immunized cohort (at the age of 6 months) assuming protective efficacy to last till 10 years and 15 years following immunization. These three strategies were compared against the counterfactual scenarios of no vaccination (with TCV) in the new-borne cohort till 15 years of age.

Trials have shown that the children immunized with single dose of TCV elicited high titres of IgG anti-Vi antibody that persisted upto 5 years in more than 80% of the immunized children [5], [6], [7]. But, there is lack of empirical evidence on the antibody titers beyond 5 years following immunization. Based on the results of a human challenge study, that also used WHO approved ‘Typbar TCV’, we considered a protective efficacy of 87% against the typhoid fever in the 1st five years of vaccine administration [7], [10]. We assumed that the efficacy of the TCV would wane by 50% (i.e., 43.5%) between 5 and 10 years and 75% (i.e., 21.75%) between 10 and 15 years following immunization with TCV. Considering this, the first vaccination scenario assumed a complete efficacy in the first 5 years of vaccine administration and no protective efficacy thereafter. In the 2nd scenario, the efficacy of vaccine was 87% in the first five years of the administration, but it reduced to 43.5% during the age of 5–10 years and no protective efficacy thereafter. Lastly, in the 3rd scenario, the vaccine efficacy was assumed to be 87%, 43.5% and 21.75% in the age of 0–5 years, 5–10 years and 10–15 years respectively.

The typhoid vaccine was assumed to be integrated alongside the routine immunization through the existing health system. Percentage of fully immunized children in the age group of 12–23 months in the urban (64%) and rural (61%) areas as estimated in the National Family Health Survey (NFHS-4) was used as the coverage for typhoid vaccination (Table 1) [33]. We modelled the protective effect of indirect or herd immunity among the unvaccinated children in the vaccinated arm of the study, that were missed out and not immunized with TCV based on the existing coverage levels of immunization in urban and rural areas. The level of herd protection against typhoid infection was assumed to be 44%, which was reported in a cluster randomized trial undertaken for assessing the effectiveness of Vi polysaccharide typhoid vaccine in India [34].

### 2.5 Cost of vaccination and typhoid treatment

Cost of vaccination included both the price of vaccine as well as its service delivery cost. The price per dose of ₹ 108 ($ 1.50) announced by Bharat Biotech for the Global Alliance for Vaccine Initiative (GAVI) supported countries was assumed in the present analysis [10]. Further, as we have assumed that the TCV will be delivered through the existing health facilities of India, we used estimates from ‘National Health System Cost Database of India (NHSCD)’ [35]. for assessing the vaccine delivery cost. NHSCD contains cost of various health services (including the vaccine delivery cost) based on data collected from 100 sub-centres (SC), 33 primary health centres (PHC), 19 community health centres (CHC) and 19 district hospitals (DH) from 6 states of India. The unit cost of vaccine delivery included the opportunity cost of human resource time involved in the vaccination, capital cost (building/space and equipment), consumables (syringes, safety boxes, needles, cotton, etc.), vaccine storage (e.g., cold chain) and its transport. We assumed that the current capacity of vaccine storage would be sufficient and no additional space or equipment would be required for its storage. Overall, the service delivery cost was estimated to be ₹ 144 ($ 2.0) per dose.

The health system treatment cost incurred of typhoid illness in the public health facilities at primary/secondary and tertiary level were derived using cost estimates from the NHSCD and the study on Cost of Health Services in India (CHSI) [35], [36]. The NHSCD provides facility specific and specialty specific mean cost of an outpatient consultation and per bed day for an inpatient stay at primary and secondary level of health facilities. Similarly, CHSI study provides mean unit cost for outpatient and inpatient care for the medicine department based on cost data collection from 52 tertiary care facilities across 13 Indian states. These mean cost values all capital and recurrent inputs included in the delivery of health care that were estimated through mixed top-down and bottom-up economic costing methods [36], [37].

For deriving the health system cost of uncomplicated typhoid cases, we used information from the standard treatment guidelines (STG) [28], [29] on the type and quantity of various diagnostics, drugs and consumables utilized for treating typhoid illness (below 15 years of age) on an outpatient basis and calculated its cost by multiplying the quantity of each of these items with its price as obtained from public procurement agencies from India [35]. This cost was then used to replace the cost incurred on diagnostics, drugs and consumables from the mean costs reported in the NHSCD and CHSI study, to convert these facility or specialty specific unit costs into typhoid specific unit cost (Table 2). In addition to heath system cost, the patient level out of pocket expenditure (OOPE) incurred during an outpatient visit on travel, boarding/lodging and other direct non-medical expenses at public level health facilities was assessed from NSS-71st round [38]. As NSS data does not capture typhoid specific OOPE, we used average expenditure incurred on all fever cases.

**Table 2:** Cost of typhoid treatment.

**Type of cost**	**Level of health care facility**	**Unit cost per service (₹)**	
		**Cost per outpatient consultation (SE)**	**Cost per bed-day cost for inpatient care (SE)**
**Health system cost**	Primary health centre	258 (53)	–
	Community health centre	268 (55)	1015 (207)
	District hospital	352 (72)	983 (201)
	Tertiary care facility	520 (106)	878 (179)
**Out of pocket expenditure(Urban ; Rural)**	Primary health centre/Community health centre/Urban Dispensary	78 ; 205 (20 ; 36)	769 (59)
	District hospital/Tertiary care hospital	130 ; 326 (24 ; 52)	1137 (159)
	Private clinic	641 ; 548 (26 ; 16)	–
	Private hospital	1076 ; 1090 (83 ; 79)	2622 (115)

₹: Indian Rupees.

For deriving the cost incurred on the treatment of a severe typhoid case, mean per bed-day cost for inpatient care at secondary and tertiary care public health facilities as reported from NHSCD and CHSI study were directly used in addition to OOPE incurred by the patients during the inpatient stay (Table 2). As the estimates on typhoid specific patient level OOPE incurred on inpatient care at the level of secondary and tertiary care public health facilities was available from NSSEFI study [39], we directly used mean health system cost reported for inpatient care from NHSCD and CHSI study (without any adjustment for typhoid specific management) to avoid any duplication of costs incurred on diagnostics, drugs and consumables. Lastly, the cost of complications was assessed by calculating the additional expenditure incurred on diagnostics, drugs and consumables (or time taken for surgical treatment) required for the management of various complications as per STGs [40], [41] and adding it to the inpatient cost incurred on typhoid cases without complications separately for DH and tertiary care facility (eAppendix: Supplementary material - Tables S3 to S6 and S8).

In the case of the private sector cost of treatment, we used average OOPE estimates incurred on outpatient care in private clinics and hospitals for all fever cases (below 15 years of age) as per NSS-75th round (Table 2) [32]. The reported OOPE incurred on hospitalization (without complications) due to typhoid illness in private sector hospitals as estimated in NSSEFI study was used [39]. For assessing the OOPE with complications, additional cost of medications and diagnostics (as per STGs) was added to the mean OOPE on hospitalization incurred by patients without any complications (eAppendix: Supplementary material- S7 and S8 table). As the OOPE expenditure data from NSSEFI study was for the year 2019, all the unit costs from other databases (NHSCD) and surveys (CHSI and NSS) used in the present study, were inflated to the year 2019 based on the GDP deflator indices for India [42]. All costs are reported in the Indian Rupees (₹).

Indirect cost inclusive of wage loss during the inpatient stay as well as productivity losses due to premature mortality was also included in the analysis. Wage loss of the parents or caregiver during the duration of inpatient stay (estimated in hours) was assessed from NSSEFI study [39]. Average wage loss of the parent or caregiver in public sector facility and private hospital was ₹ 2968 (based on an average of 136 person-hours of time lost) and ₹ 8045 (based on average of 161 person-hours of time lost) respectively. Productivity cost lost due to premature death was calculated by multiplying the expected working lifetime of an individual (from 18 years to 60 years) lost due to premature death with an average per capita income earned in India [43].

### 2.6 Quality of life

Primary data was collected for assessing the quality of life (QoL) of patients hospitalized for typhoid illness. 109 typhoid infected hospitalized children were recruited from health care facilities across 7 states of India and interviewed using EQ-5D-5L tool. The utility value for EQ-5D-5L health states were obtained based on recently generated tariff values for India [44]. The mean utility value for patients in the uncomplicated stage was estimated by applying a relative reduction factor on the observed utility value from our primary data. The reduction factor was derived from published studies between typhoid patients hospitalized and those being treated an outpatient basis [45], [46].

### 2.7 Sensitivity analysis

Multivariable probabilistic sensitivity analysis (PSA) was carried out to account for parameter uncertainty [47]. Under PSA, specific distributions were assigned to each of input parameters based on its nature. Gamma distribution was used for cost parameters and beta distribution for epidemiological rates or proportions and utility values. Ranges of disease and vaccine specific parameters are mentioned in Table 1 and Table 2. Finally, after assigning both the distribution and range to each of the parameter values, 999 Monte Carlo simulations were run, from which a median value of incremental cost effectiveness ratio (ICER) along with 2.5th and 97.5th percentile were reported.

A few scenario analyses were also undertaken. Firstly, the results are presented for each the 3 vaccination scenarios separately for urban and rural areas. Secondly, results are presented with or without inclusion of indirect costs. Thirdly, a univariate sensitivity analysis was undertaken in which the effect of indirect protection provided by herd immunity was not included in the analysis. Fourthly, based on higher incidence of typhoid in the age group of 5–10 years in urban settings, an additional scenario of specifically introducing typhoid vaccination at 5th year of age (school going children) was evaluated. Further, instead of assuming a waning efficacy rate of 50% for typhoid vaccine beyond 5 years, a more favourable scenario considering a waning efficacy rate of 10% and 20% in the age group of 5–10 years and 10–15 years following immunization was assumed. A worse-case scenario was also included, in which a 75% reduction in the efficacy of TCV in the age group of 5–10 years and zero protection rate thereafter was considered. Lastly, threshold analyses were undertaken to ascertain the probability of TCV to be cost effective (at 1 × GDP per capita) with changes in the price of TCV and incidence of typhoid infection in urban areas.

## 3 Results

### 3.1 Urban settings

We found that introduction of TCV results in 17%, 31% and 36% reduction in the number of typhoid cases and deaths assuming that the protective effect lasts for 5 years, 10 years and 15 years respectively (Panel A; Table 3). This reduction in disease burden led to the gain of 54 (33–81) to 82 (51–120) LYs and 67 (41–98) to 105 (66–151) QALYs per 100,000 new-borns. With exclusion of indirect costs, the incremental cost (in thousands) was ₹ 10,138 (4453–16,347), ₹ 6016 (−592 to 12,786) and ₹ 4647 (−2334 to 11,764) for scenario 1, 2 and 3 respectively. Further, when indirect costs are included, all the 3 scenarios were cost saving (Table 3 and eAppendix: Supplementary material- Table S9).

**Table 3:** Summary of model predicted incremental cost and health outcomes in the newly borne cohort of 100,000 children following vaccination as compared to no vaccination in India.

**Incremental outcomes**	**Vaccination strategies (as compared to no vaccination)**		
	**Scenario 1: Protective efficacy of 5 years**	**Scenario 2: Protective efficacy of 10 years**	**Scenario 3: Protective efficacy of 15 years**
***Panel A: Urban settings***			
Typhoid cases averted (% decrease)	2057 (17)	3668 (31)	4284 (36)
Typhoid deaths averted (% decrease)	5.7 (17)	10.2 (31)	12 (36)
Life years gained	54 (33–81)	79 (49–117)	82 (51–120)
QALY gained	67 (41–98)	100 (62–144)	105 (66–151)
Incremental cost in ₹ 1000 s (excluding indirect costs)	10,138 (4453–16,347)	6016 (−592 to 12,786)	4647 (−2334 to 11,764)
Incremental cost in ₹ 1000 s (including indirect costs)	−4540 (−16,135 to 4029)	−19,876 (−36,512 to −6703)	−25561 (−43,773 to −10,949)
Incremental cost (₹) per QALY gained (excluding indirect costs)	151,346 (54,730–307,975)	61,710 (−5250 to 163,283)	45,188 (−17,069 to 141,093)
Incremental cost (₹) per QALY gained (including indirect costs)	−69,293 (−171,665 to 86,632)	−200,336 (−274,441 to −99,324)	−241,986 (−317,246 to −144,122)
***Panel B: Rural settings***			
Typhoid cases averted	98 (19)	153 (30)	180 (36)
Typhoid deaths averted	0.35 (19)	0.55 (30)	0.65 (36)
Life years gained	3.31 (2–4.9)	4.39 (2.75–6.30)	4.56 (2.87–6.52)
QALY gained	3.92 (2.45–5.58)	5.30 (3.36–7.46)	5.59 (3.58–7.86)
Incremental cost in ₹ 1000 s (excluding indirect costs)	14,882 (10,214–20,772)	14,734 (10,096–20,622)	14,672 (10,041–20,564)
Incremental cost in ₹ 1000 s (including indirect costs)	13,991 (9453–19,796)	13,367 (8700–19,187)	13,058 (8394–18,896)
Incremental cost (₹ 1000 s) per QALY gained (excluding indirect costs)	3796 (3383–6903)	2787 (1699–4988)	2630 (1597–4656)
Incremental cost (₹ 1000 s) per QALY gained (including indirect costs)	3574 (2057–6691)	2524 (1451–4733)	2340 (1316–4370)

*QALY: Quality adjusted life years; ₹: Indian Rupees.

The incremental cost per QALY gained with TCV (excluding the indirect costs) was ₹ 151,346 (54,730–307,975), ₹ 61,710 (−5250 to 163,283) and ₹ 45,188 (−17,069 to 141,093) with scenarios 1, 2 and 3 respectively. With inclusion of indirect costs, all the 3 scenarios were cost saving with ICER per QALY gained of ₹ −69,293 (−171,665 to 86,632), ₹ −200,336 (−274,441 to −99,324) and ₹ −241,986 (−317,246 to −144,122) for scenario 1, 2 and 3 respectively. Lastly, based on the interpretation of cost-effectiveness acceptability curves, it was seen that, when excluding the indirect costs, the probability of scenario 1, 2 and 3 to be cost effective was 47.5%, 94% and 98% respectively at willingness to pay (WTP) threshold value of GDP per capita of India. (eAppendix: Supplementary material- Fig. S1). Similarly, with inclusion of indirect costs there was 99% probability of scenario 1 to be cost-effective, while scenario 2 and 3 to be certainly (100%) cost effective at a threshold of per-capita GDP (eAppendix: Supplementary material- Fig. S2)

### 3.2 Rural settings

In urban settings, introducing TCV vaccine in rural areas in a cohort of 100,000 new-borns would result in 19% to 36% reduction in typhoid cases and deaths, along with the gain of 3.92 (2.45–5.58) to 5.59 (3.58–7.86) QALYs at an incremental cost (inclusive of indirect costs) of ₹ 13,058 (8394–18,896) to ₹ 13,991 (9453–19,796) thousand across the 3 vaccination scenarios (Panel B of table 3; eAppendix: Supplementary material - Table S9). This resulted in an ICER (incremental cost per QALY gained; inclusive of indirect costs) of ₹ 2340 (1316–4370) to ₹ 3574 (2057–6691) thousand among the 3 vaccination scenarios.

### 3.3 Sensitivity analysis

When the effect of indirect protection provided by herd immunity was not included in the analysis, there was lesser reduction of 14% to 30% in typhoid incidence and mortality, and ICER (per QALY gained; excluding the indirect costs) was increased to ₹ 202,139, ₹ 97,087 and ₹ 79,611 in scenarios 1, 2 and 3 respectively in the urban population. However, with inclusion of indirect costs, all the 3 scenarios were still cost saving (eAppendix: Supplementary material - Tables S10 and S11). Further, in a scenario when TCV was assumed to be delivered at 5 years of age (and assuming the vaccine efficacy to last for 5 years) in urban settings, there was a 28% relative reduction in the incidence of typhoid infection along with gain of 69 QALYs per 100,000 new-borns as compared to no vaccination. This resulted in an incremental cost of ₹ −228,551 and ₹ 115,908 (cost-saving) per QALY gained with and without inclusion of indirect costs (eAppendix: Supplementary material - Table S12). Furthermore, in another scenario that considered efficacy to wane by of 10% and 20% for TCV in the age group of 5–10 years and 10–15 years respectively, ICER (without indirect costs) decreased to ₹ 22,124 and ₹ −9113 (cost saving) per QALY gained with outcomes assessed over 10 years and 15 years of duration of protective efficacy respectively (eAppendix: Supplementary material - Table S13). Lastly, when considering 75% reduction in the efficacy of TCV in the age of 5–10 years of age and zero protection rate thereafter, ICER (per QALY gained) came out to be ₹ 93,401 and ₹ −151,808 (cost-saving) with and without exclusion of indirect costs respectively (eAppendix: Supplementary material - Table S14).

**Fig. 2:**
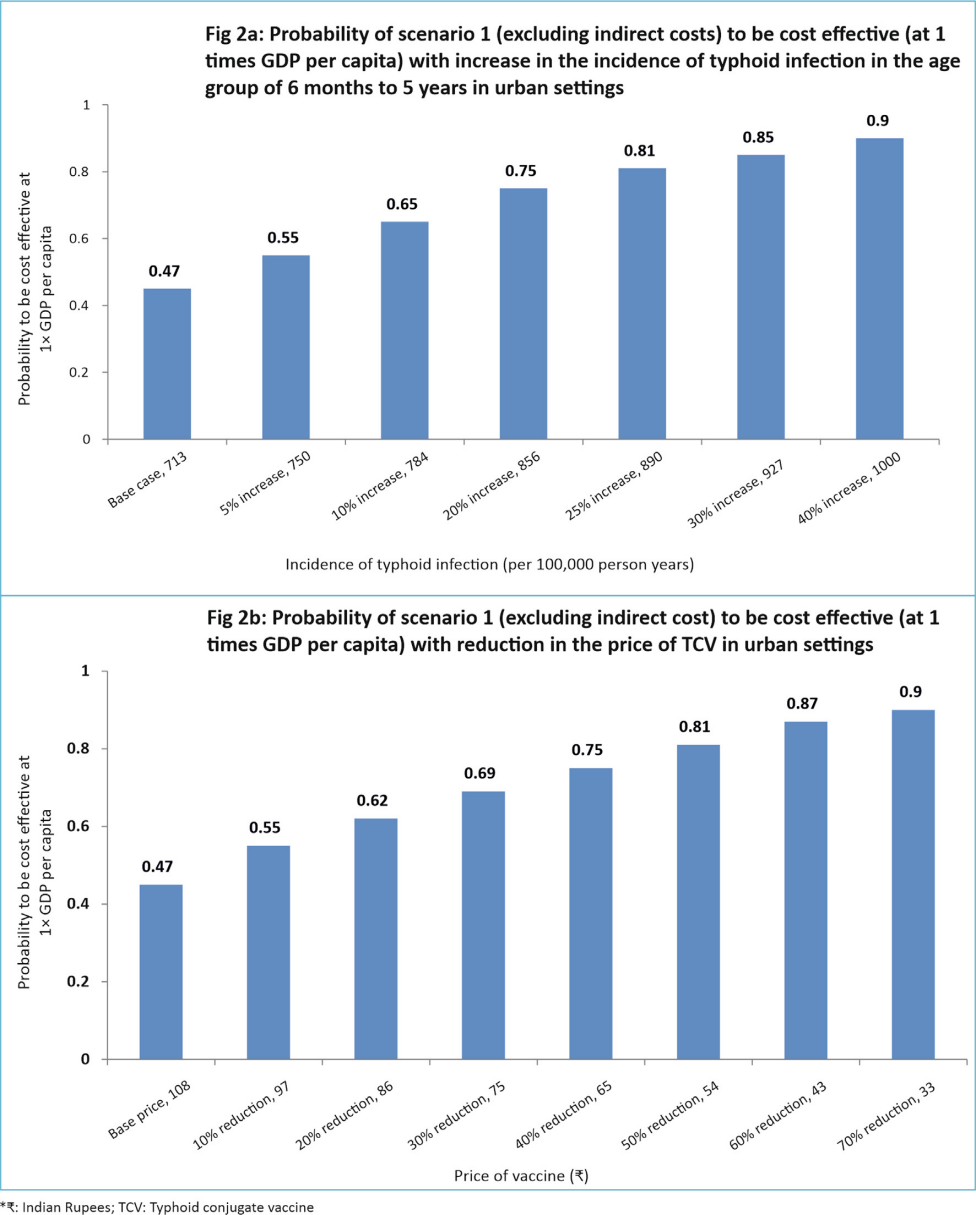
Threshold analysis.

Based on the threshold analysis, it was seen that when the price of TCV is reduced to ₹ 33, there is 90% probability of scenario 1 (excluding the indirect cost) to be cost effective in urban settings (Fig. 2). Similarly, when the incidence of typhoid infection increases to 1000 per 100,000 child years in the age group of 6 months to 5 years in urban areas, there is 90% probability of scenario 1 to be cost effective after exclusion of indirect costs.

## 4 Discussion

We assessed the cost-effectiveness of introducing TCV vaccination separately for urban and rural population of India using a decision analytic model under a variety of assumptions regarding costs, duration of protective effect, price, etc. Our analysis demonstrated a decrease in the incidence and mortality of typhoid infection ranging from 17% to 36%, which translated to a gain of 4 to 105 QALYs per 100,000 children across different vaccination scenarios as compared to no vaccination in India. Our study findings show that if societal perspective is considered (i.e., inclusive of indirect costs), TCV introduction is a cost saving strategy in the urban settings. However, if indirect costs are excluded from the analysis, TCV is not cost effective for scenario 1 (i.e., when considering vaccine efficacy to last for 5 years), but is still cost effective for the remaining 2 scenarios (with outcomes assessed over 10 years and 15 years of duration of protective efficacy respectively). Lastly, the results show that TCV introduction is not a cost effective strategy in rural settings of India.

A previous study on typhoid vaccination showed that in countries with 300 or more cases of typhoid infection per 100,000 person-years, immunization with TCV is likely to be cost effective [19]. Similarly, an economic evaluation from India using incidence data from the urban slums of Delhi and Kolkata predicted routine immunization with TCV to be cost-saving in Delhi and very cost effective in Kolkata [18]. Both these studies had assumed duration of vaccine induced immunity to be ≥ 10 years and had used more than a decade old incidence rates from only 2 sites across India. Furthermore, the cost in previous studies is dated and did not include cost of the caregiver’s time, transportation costs and indirect costs [48], [49]. Our study on similar lines with previous studies shows TCV to be cost effective, but only in urban settings of India. But in comparison to these studies, the present analysis is based on a recent nationally representative estimates of incidence rates, treatment cost (inclusive of direct, non-direct and indirect cost), along with the use of more conservative approach with regards to duration of efficacy for TCV beyond 5 years, presents more robust findings and segregated results comprising of analysing the effect of TCV introduction separately for urban and rural settings as well as with and without inclusion of indirect costs.

The Indian guidelines for undertaking of economic evaluations by Health Technology Assessment Board of India specifically states to not include indirect costs in the base case analysis of a cost effectiveness study [21]. However, it encourages presentation of a scenario by including the effect of indirect costs as a part of sensitivity analysis. We followed these guidelines and accordingly examined the effect of with and without inclusion of indirect costs on the outcome of the study. Inclusion of indirect costs certainly had a major impact and made TCV more cost effective. It was seen that indirect costs constituted around 70%−80% of the total cost of illness incurred on the typhoid both in the urban and rural areas. This finding is also similar to the results of a systematic review on ‘Economic Evidence of Typhoid Fever and Typhoid Vaccines’ that reported the share of indirect cost to be in the range of 80% to 89% of the total treatment cost [50]. The reason for higher indirect costs was mainly due to the huge productivity losses incurred because of the premature mortality occurring in the young age of 0–15 years caused by the typhoid infection. To be precise, productivity loss due to premature deaths constituted of more than 88% of the total indirect costs. Because of these savings arising from the reduction in productivity losses, as a result of the reduction in the premature mortality, following typhoid vaccination (as compared to no vaccination) lead to TCV being more cost effective.

The difference in the outcome between urban and rural settings is perhaps the most interesting finding of the paper. Incidence of typhoid infection was one of the important input parameters that had a major impact on the outcome of the present analysis. The incidence of typhoid infection in the rural areas (i.e., 35 per 100,000 child years), as estimated by NSSEFI study, is reported to be around 1/20th of the infection in the urban areas within the age group of 0–5 years [20]. Thus, a clear policy recommendation from the present analysis is to specifically target introducing the TCV in the urban slums and urban areas of the country.

The results of the present study could strengthen the policy level decisions (on the introduction of TCV) undertaken by NTAGI as well as the Ministry of Health at Central and State levels. NTAGI provides broader recommendation on the utility of the given vaccine to the Ministry of Health [14] and several choices for implementation of the program rests with the Central and State Governments. These choices could include the coverage of free immunization program in rural versus urban area, target age group to be vaccinated, nature of service delivery (routine versus school based). Several of these questions are answered based on the evidence generated from the present analysis. Further, as the present analysis was specifically focussed on Indian context, it is difficult to adapt the evidence generated as part of present study to other settings in the absence of actual data from the other typhoid endemic countries. However, the present model findings may be used along with other novel methods like that of adaptive or rapid HTA, as reported elsewhere [51], to address the issue of generalizability in other countries.

### 4.1 Strengths and limitations

The use of standard EQ-5D-5L tool for derivation of utility values based on recently generated tariff value set from India [44], [52], is one of the major strength of the study. This value set of India has been generated based on data collected from 2409 participants across 5 states of India. Previously undertaken economic evaluations from India had either utilized Thailand specific value set to generate utility index values or had directly used QoL estimates from studies conducted in other countries [53], [54], [55]. The use of local preferences in the estimation of QoL values adds credibility in the assessment of QALYs. Moreover, the use of real world estimates of treatment seeking behaviour (for children ≤ 15 years of age) and vaccine coverage based on latest nationwide NSSO and NFHS surveys provides strength to the study [32], [33]. Furthermore, as the present analysis assumed to integrate delivery of the TCV along with routine immunization, the use of real world estimate on the cost of vaccine delivery that also took into account the existing capacity and prevailing levels of efficiency across the health facilities from 6 states of India, tends to accurately capture the total cost of immunization with TCV [35].

We do acknowledge that that the standard EQ-5D-5L tool is for adults. We could have used the EQ-5D-Y instrument [56], the standard tool for estimating Qol in children, however, it would not have been possible for us to calculate QALYs, as no value-set for EQ-5D-Y is available for India. Considering the dissimilarity between the use of EQ-5D-Y and EQ-5D-3L, a study investigated the difference in valuations of health states by using these 2 tools and reported that the observed utility values were on a lower side by 6% (absolute difference) with the use of EQ-5D-3L compared to ED-5D-Y in children [57]. The implication is that if we would have used EQ-5D-Y in place of EQ-5D-5L (although the evidence presented above pertains to 3L, not 5L), the observed utility scores might have been on the higher side, which further could have reduced the magnitude of health gain and increased the ICER value. So, we did a sensitivity analysis and increased the utility values of each of the health state by 6% (absolute increase) and observed that there was no difference in the direction of the results (eAppendix: Supplementary material - Table S15). Secondly, for most of our utility values, we had also varied the base values from 5% to 18% (on the higher side) in probabilistic sensitivity analysis that also balances the effect of lower valuation of utility scores using EQ-5D-5L.

The inclusion of herd immunity made the present analysis more realistic, by estimating, in addition to the direct protection from vaccination, actual reduction in typhoid cases by incorporating typhoid infections averted among the unvaccinated children also. If the indirect protection from herd immunity was not included in the analysis, there would have been 4% to 10% relatively lesser reduction in the incidence of typhoid infection as well as mortality and 2.6% to 8% higher cost of illness among the 3 vaccination scenarios in the urban settings of the country (eAppendix: Supplementary material - Tables S10 and S11). However, we acknowledge that herd immunity can have manifold effects in terms of reduction in the typhoid incidence among adults as a result of vaccination among children. We did not measure this effect among adults (i.e., >15 years of age) in the present study. Considering the fact, that the incidence of typhoid infection among those above 15 years of age, which is reported to be around 1/12th of the infection among less than 15 years of age [34], we can easily assume that reduction in typhoid incidence due to herd immunity among adults will contribute to a minor proportion as compared to the reduction observed among children <15 years of age.

Our analysis was limited by the paucity of literature on duration of efficacy of typhoid vaccine beyond 5 years of its administration. We assumed a range of different scenarios to assess the effect of changing levels of protective efficacy of TCV beyond 5 years of vaccination on the outcome of the study. We considered a conservative approach (i.e., base case), favourable approach and a worse-case scenario as well as varied the efficacy rate of vaccine in the PSA. The outcomes of all these scenarios, even with a strict reduction in the efficacy of the TCV by 75% in the age group of 5–10 years (eAppendix: Supplementary material - Table S14), were in the similar direction, that showed TCV to be cost effective in beyond 5 years of age.

Despite evidence in favour of reduction in antimicrobial resistance with introduction of vaccination [58], we did not factor it in our analysis. Although it has been seen that multidrug-resistant (MDR) typhoid is responsible for increased complications, case fatality [59], [60] and thereby expenditure, we excluded it from the analysis as the guidelines for management of severe enteric fever in children issued by Indian Academy of Paediatrics and as well as National Health Mission (Ministry of Health and Family Welfare, Government of India), already recommends use of ceftriaxone or cefotaxime for 14 days in both sensitive as well as multi drug resistant cases [28], [29]. Moreover, a study undertaken to determine the antibiotic prescription practices in children attending a tertiary care hospital also showed that ceftriaxone and cefixime were first line of antibiotic treatment for typhoid fever being used in outpatient as well as hospitalized patients [61].

The objective of the present study was to assess the cost effectiveness of TCV delivered along with routine immunization schedule in public health facilities of India. The intervention scenario is compared against a counterfactual of routine care which is no typhoid vaccination. This is justified based on the fact that the current coverage rate of typhoid vaccination (using either of the available typhoid vaccines) is only around 3%, and the entire share of this immunization is through the private sector hospitals and clinics of India [62]. Among the three available typhoid vaccines, we modelled the costs and effects of TCV in view of the WHO recommendations in its favour [9]. Moreover, since the efficacy of TCV is highest and its cost is lower than ViPS, we consider the choice of TCV as intervention scenario appropriate [63].

## 5 Conclusion

Based on the current incidence and treatment cost of typhoid in India, introduction of TCV is a cost saving strategy in urban India from a societal perspective. In rural India, introduction of TCV is not a cost-effective strategy. Lastly, with TCV coming out to be more cost effective in the longer run i.e., up to 5–10 years and 10–15 years post vaccination as compared to available evidence of efficacy up to 5 years of vaccination, we recommend that the focus of on-going/future research in the field of immunogenicity and efficacy of typhoid vaccination should be on assessing the efficacy and its long term benefits beyond 5 years of vaccination.

## Funding

Bill & Melinda Gates Foundation (OPP115935) to Christian Medical College Vellore. The funders had no role in study design, data collection and analysis, decision to publish, or preparation of the manuscript.

## Declaration of Competing Interest

The authors declare that they have no known competing financial interests or personal relationships that could have appeared to influence the work reported in this paper.
