# Supplementary material for: Cost effectiveness of typhoid vaccination in India
Source: Vaccine. 2021 Jul 5;39(30):4089–98. doi: 10.1016/j.vaccine.2021.06.003 (PMC8256879; doi:10.1016/j.vaccine.2021.06.003)
Supplement: Supplementary data 1 [file mmc1.docx]

**Appendix A: Supplementary material**

**Fig S1: Cost effectiveness acceptability curves without inclusion of indirect cost for urban settings**

**
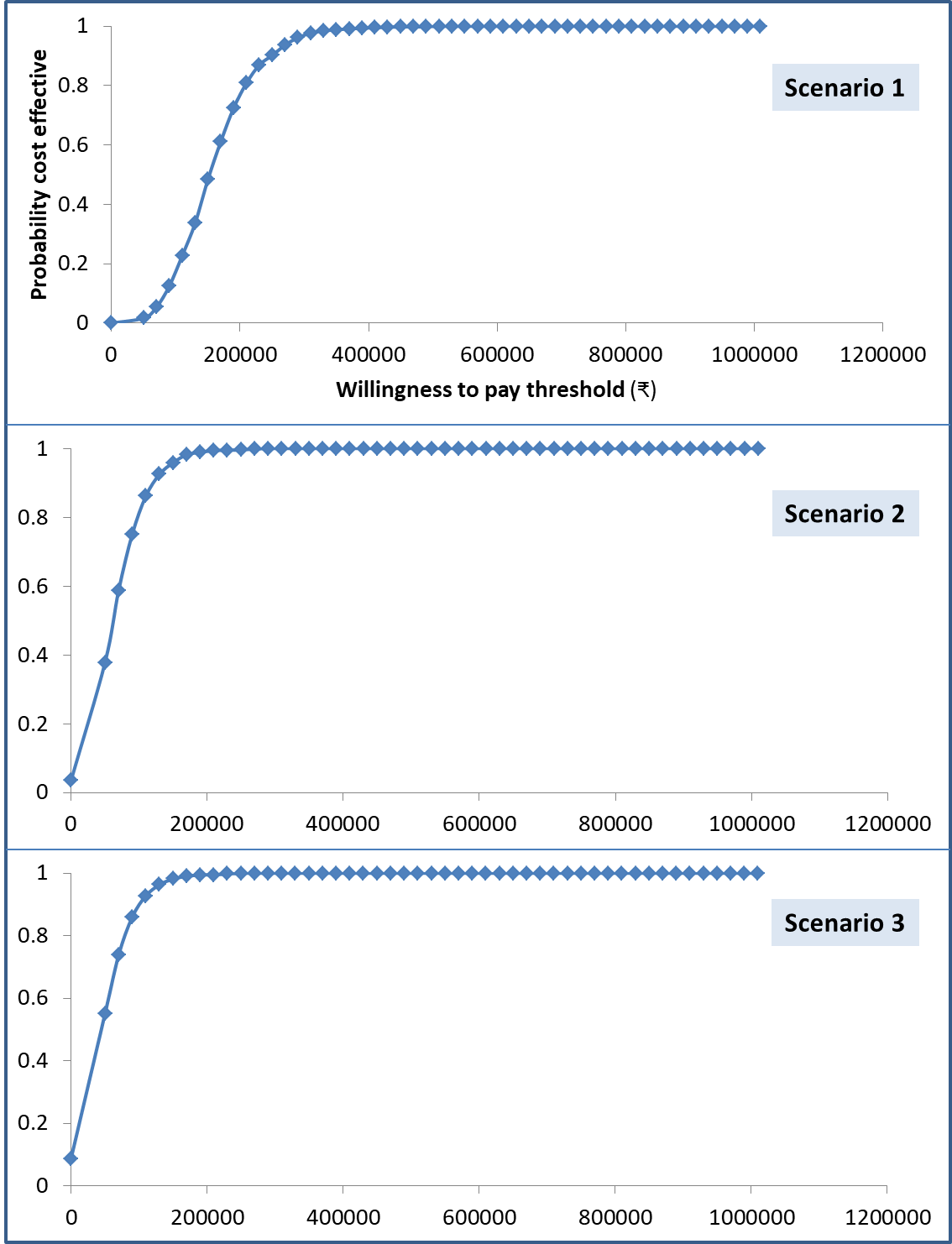
**

**Fig S2: Cost effectiveness acceptability curves with inclusion of indirect cost for urban settings**

**
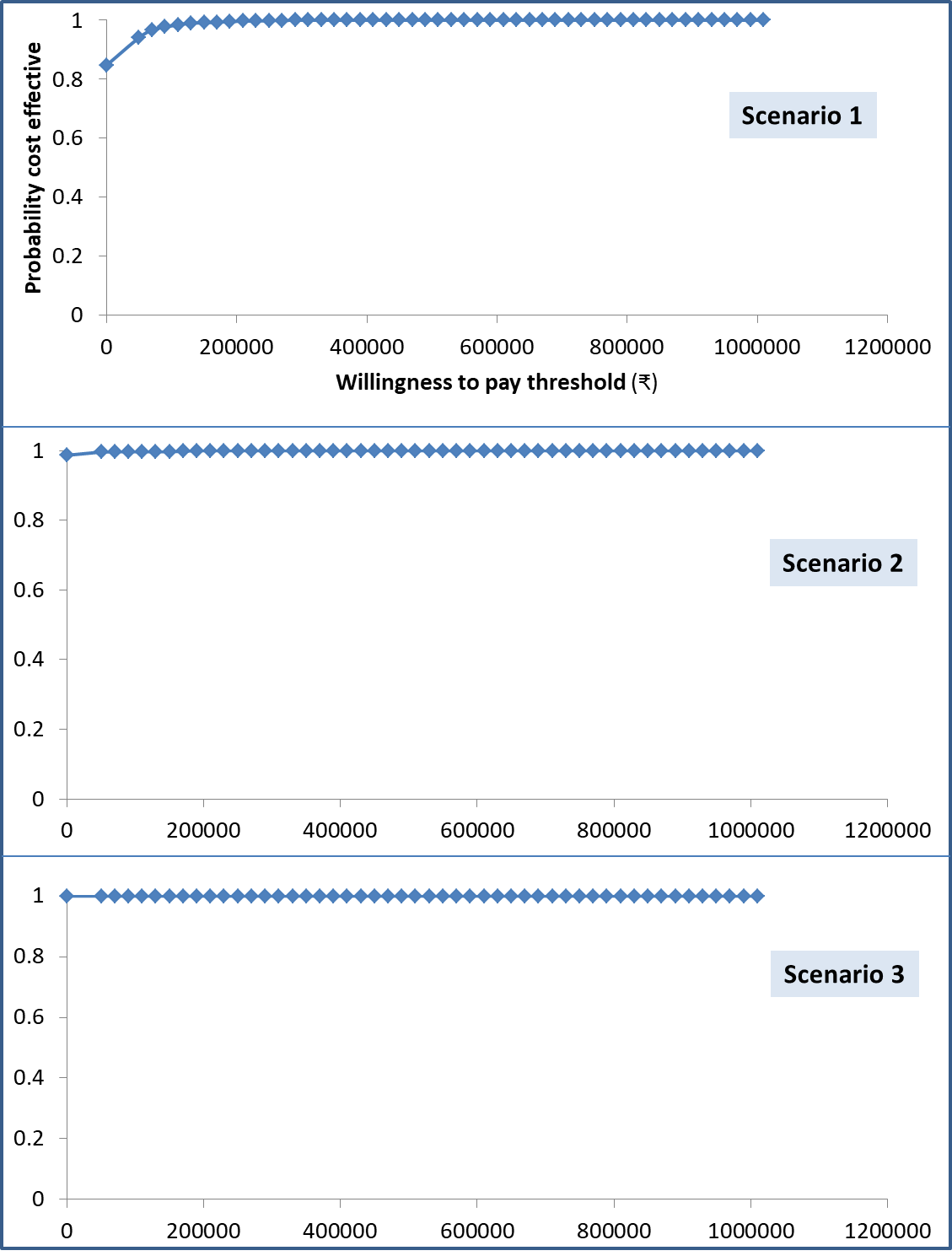
**

**S1 Table: Age specific all-cause mortality rates of India**

| **Age group** | **Annual mortality rates (per person)** | |
| --- | --- | --- |
|  | **Urban settings** | **Rural settings** |
| **Less than 1 years** | 0.02572 | 0.04392 |
| **1 – 4 years** | 0.00256 | 0.00784 |
| **5 – 9 years** | 0.0025 | 0.00384 |
| **10 – 14 years** | 0.00235 | 0.00329 |
| **15 – 19 years** | 0.00374 | 0.00494 |

**S2 Table: Treatment seeking pattern of typhoid infected patients**

| **Severity level** | **Type of facility** | **Level of health facility** | **Utilization pattern** | |
| --- | --- | --- | --- | --- |
|  |  |  | **Urban** | **Rural** |
| Uncomplicated | Public | PHC/CHC/Urban Disp. | 6% | 16% |
|  |  | District hospital | 11% | 8% |
|  |  | Tertiary care hospital | 5% | 3% |
|  | Private | Private Doctor/clinic | 57% | 57% |
|  |  | Private hospital | 21% | 16% |
| Severe | Public | CHC/Urban Disp. | 4% | 12% |
|  |  | District hospital | 23% | 28% |
|  |  | Tertiary care hospital | 10% | 12% |
|  | Private | Private hospital | 63% | 48% |
| Complicated | Public | District hospital | 26% | 37% |
|  |  | Tertiary care hospital | 11% | 15% |
|  | Private | Private hospital | 63% | 48% |

*PHC: Primary health center; CHC: Community health center; Disp.: Dispensary

**S3 Table: Health system cost of complications incurred at the level of district hospital**

| **Type of complication** | **Per bed day cost in ₹** | | |
| --- | --- | --- | --- |
|  | **0-2 years** | **3-5 years** | **5-15 years** |
| Hepatitis | 1019 | 1091 | 1162 |
| Encephalopathy | 987 | 993 | 1001 |
| Gastro-intestinal bleeding | 985 | 985 | 985 |
| Renal Impairment | 1041 | 1109 | 1227 |
| Hemodynamic shock | 999 | 1002 | 1013 |
| Myocarditis | 1021 | 1058 | 1119 |
| Pneumonia | 1022 | 1109 | 1206 |
| Urinary tract infections | 1056 | 1156 | 1280 |
| Osteomyelitis | 1070 | 1208 | 1382 |

₹: Indian Rupees

**S4 Table: Health system cost of complications incurred at the level of tertiary care hospital**

| **Type of complication** | **Per bed day cost in ₹** | | |
| --- | --- | --- | --- |
|  | **0-2 years** | **3-5 years** | **5-15 years** |
| Hepatitis | 914 | 986 | 1057 |
| Encephalopathy | 882 | 888 | 896 |
| Gastro-intestinal bleeding | 880 | 880 | 880 |
| Renal Impairment | 936 | 1004 | 1122 |
| Hemodynamic shock | 894 | 897 | 908 |
| Myocarditis | 916 | 953 | 1014 |
| Pneumonia | 917 | 1004 | 1101 |
| Urinary tract infections | 951 | 1051 | 1175 |
| Osteomyelitis | 965 | 1103 | 1277 |

₹: Indian Rupees

**S5 Table: Out of pocket expenditure on complications incurred at the level of district hospital**

| **Type of complication** | **Per bed day cost in ₹** | | |
| --- | --- | --- | --- |
|  | **0-2 years** | **3-5 years** | **5-15 years** |
| Hepatitis | 836 | 969 | 1102 |
| Encephalopathy | 777 | 788 | 802 |
| Gastro-intestinal bleeding | 772 | 772 | 772 |
| Renal Impairment | 877 | 1004 | 1222 |
| Hemodynamic shock | 799 | 805 | 825 |
| Myocarditis | 840 | 908 | 1021 |
| Pneumonia | 842 | 1003 | 1183 |
| Urinary tract infections | 905 | 1089 | 1321 |
| Osteomyelitis | 931 | 1187 | 1510 |

₹: Indian Rupees

**S6 Table: Out of pocket expenditure on complications incurred at the level of public sector tertiary care hospital**

| **Type of complication** | **Per bed day cost in ₹** | | |
| --- | --- | --- | --- |
|  | **0-2 years** | **3-5 years** | **5-15 years** |
| Hepatitis | 1204 | 1337 | 1470 |
| Encephalopathy | 1145 | 1156 | 1170 |
| Gastro-intestinal bleeding | 1140 | 1140 | 1140 |
| Renal Impairment | 1245 | 1372 | 1590 |
| Hemodynamic shock | 1167 | 1173 | 1193 |
| Myocarditis | 1208 | 1276 | 1389 |
| Pneumonia | 1210 | 1371 | 1551 |
| Urinary tract infections | 1273 | 1457 | 1689 |
| Osteomyelitis | 1299 | 1555 | 1878 |

₹: Indian Rupees

**S7 Table: Out of pocket expenditure on complications incurred at the level of private hospital**

| **Type of complication** | **Per bed day cost in ₹** | | |
| --- | --- | --- | --- |
|  | **0-2 years** | **3-5 years** | **5-15 years** |
| Hepatitis | 2725 | 2930 | 3135 |
| Encephalopathy | 2634 | 2651 | 2673 |
| Gastro-intestinal bleeding | 2627 | 2627 | 2627 |
| Renal Impairment | 2789 | 2983 | 3319 |
| Hemodynamic shock | 2668 | 2678 | 2709 |
| Myocarditis | 2731 | 2836 | 3010 |
| Pneumonia | 2734 | 2982 | 3260 |
| Urinary tract infections | 2832 | 3115 | 3471 |
| Osteomyelitis | 2871 | 3265 | 3762 |

₹: Indian Rupees

**S8 Table: Cost of illeal perforation surgery**

| **Type of health facility** | **Cost in ₹*** |
| --- | --- |
| District hospital^@^ | 19,468 |
| Tertiary care hospital^@^ | 24,665 |
| Private hospital^#^ | 34,443 |

*This cost includes the expenditure incurred on surgery inclusive of inpatient stay of 7 days

^@^Includes both health system cost and out of pocket expenditure

^#^Estimated as OOPE incurred in private hospitals

₹: Indian Rupees

**S9 Table: Summary of model predicted absolute cost and health outcomes in the newly borne cohort of 100,000 children followed till 5, 10 and 15 years with or without vaccination in India**

| **Absolute outcomes** | | **Counterfactual: No vaccination** | **Intervention: Vaccination strategies** | | | |
| --- | --- | --- | --- | --- | --- | --- |
|  |  |  | **Scenario 1: Protective efficacy of 5 years** | **Scenario 2: Protective efficacy of 10 years** | **Scenario 3: Protective efficacy of 15 years** | |
| ***Panel A: Urban settings*** | | |  | | | |
| **Health outcomes** | Typhoid cases | 11,924 | 9829 | 8209 | | 7591 |
|  | Typhoid deaths | 33.3 | 27.5 | 23.0 | | 21.3 |
|  | Life years lived | 1,178,738 | 1,178,807 | 1,178,835 | | 1,178,838 |
|  | QALY lived | 1,178,675 | 1,178,749 | 1,178,787 | | 1,178,795 |
| **Cost in ₹ 1000s** | Health system cost | 5799 | 4662 | 3832 | | 3590 |
|  | Out of pocket expenditure | 24,248 | 19,508 | 16,237 | | 15,075 |
|  | Indirect costs | 84,417 | 69,499 | 58,018 | | 53,726 |
|  | Vaccination cost |  | 15,891 | 15,891 | | 15,891 |
|  | Total cost | 114,559 | 109,935 | 94,439 | | 88,735 |
| ***Panel B: Rural settings*** | | |  | | | |
| **Health outcomes** | Typhoid cases | 501 | 404 | 348 | 319 | |
|  | Typhoid deaths | 1.8 | 1.4 | 1.2 | 1.1 | |
|  | Life years lived | 1,154,725 | 1,154,729 | 1,154,731 | 1,154,731 | |
|  | QALY lived | 1,154,723 | 1,154,727 | 1,154,729 | 1,154,729 | |
| **Cost in ₹ 1000s** | Health system cost | 317 | 245 | 210 | 195 | |
|  | Out of pocket expenditure | 905 | 702 | 603 | 559 | |
|  | Indirect costs | 4390 | 3515 | 3029 | 2789 | |
|  | Vaccination cost |  | 15,154 | 15,154 | 15,154 | |
|  | Total cost | 5600 | 19,646 | 19,010 | 18,704 | |

*QALY: Quality adjusted life years; ₹: Indian Rupees

**S10 Table: Summary of model predicted absolute cost and health outcomes in the newly borne cohort of 100,000 children followed till 5, 10 and 15 years with or without vaccination by excluding the effect of indirect protection provided by herd immunity**

| **Absolute outcomes** | | **Counterfactual: No vaccination** | **Intervention: Vaccination strategies** | | | |
| --- | --- | --- | --- | --- | --- | --- |
|  |  |  | **Scenario 1: Protective efficacy of 5 years** | **Scenario 2: Protective efficacy of 10 years** | **Scenario 3: Protective efficacy of 15 years** | |
| ***Panel A: Urban settings*** | | |  | | | |
| **Health outcomes** | Typhoid cases | 11,924 | 10,243 | 8908 | | 8390 |
|  | Typhoid deaths | 33.3 | 28.66 | 24.90 | | 23.47 |
|  | Life years lived | 1,178,738 | 1,178,773 | 1,178,793 | | 1,178,796 |
|  | QALY lived | 1,178,675 | 1,178,719 | 1,178,746 | | 1,178,751 |
| **Cost in ₹ 1000s** | Health system cost | 5799 | 4494 | 3894 | | 3693 |
|  | Out of pocket expenditure | 24,248 | 19691 | 17058 | | 16178 |
|  | Indirect costs | 84,417 | 72,495 | 62,969 | | 59,389 |
|  | Vaccination cost |  | 15,891 | 15,891 | | 15,891 |
|  | Total cost | 114,559 | 112,781 | 100,021 | | 95,360 |
| ***Panel B: Rural settings*** | | |  | | | |
| **Health outcomes** | Typhoid cases | 501 | 422 | 378 | 356 | |
|  | Typhoid deaths | 1.8 | 1.51 | 1.35 | 1.27 | |
|  | Life years lived | 1,154,725 | 1,154,762 | 1,154,762 | 1,154,763 | |
|  | QALY lived | 1,154,723 | 1,154,759 | 1,154,760 | 1,154,761 | |
| **Cost in ₹ 1000s** | Health system cost | 317 | 243 | 217 | 206 | |
|  | Out of pocket expenditure | 905 | 726 | 648 | 614 | |
|  | Indirect costs | 4390 | 3691 | 3302 | 3110 | |
|  | Vaccination cost |  | 15,154 | 15,154 | 15,154 | |
|  | Total cost | 5600 | 20,005 | 19,512 | 19,274 | |

**S11 Table: Summary of model predicted incremental cost and health outcomes in the newly borne cohort of 100,000 children following vaccination (by excluding the effect of indirect protection provided by herd immunity) as compared to no vaccination in India**

| **Incremental outcomes** | **Vaccination strategies (as compared to no vaccination)** | | |
| --- | --- | --- | --- |
|  | **Scenario 1: Protective efficacy of 5 years** | **Scenario 2: Protective efficacy of 10 years** | **Scenario 3: Protective efficacy of 15 years** |
| ***Panel A: Urban settings*** | | | |
| Typhoid cases averted (% decrease) | 1715 (14) | 3051(26) | 3568 (30) |
| Typhoid deaths averted (% decrease) | 5 (14) | 9 (26) | 10 (30) |
| Life years gained | 45.67 | 66.09 | 68.54 |
| QALY gained | 56.34 | 84.00 | 88.87 |
| Incremental cost in ₹ 1000s (excluding indirect costs) | 11389 | 8155 | 7075 |
| Incremental cost in ₹ 1000s (including indirect costs) | -1055 | -13,815 | -18,475 |
| Incremental cost (₹) per QALY gained (excluding indirect costs) | 202,139 | 97,087 | 79,611 |
| Incremental cost (₹) per QALY gained (including indirect costs) | -18,716 | -164,463 | -207,890 |
| ***Panel B: Rural settings*** | | | |
| Typhoid cases averted | 79(16) | 123(25) | 145(29) |
| Typhoid deaths averted | (16) | (25) | 1(29) |
| Life years gained | 2.67 | 3.54 | 3.68 |
| QALY gained | 3.16 | 4.27 | 4.51 |
| Incremental cost in ₹ 1000s (excluding indirect costs) | 15136 | 15032 | 14,986 |
| Incremental cost in ₹ 1000s (including indirect costs) | 14435 | 13941 | 13,704 |
| Incremental cost (₹ 1000s) per QALY gained (excluding indirect costs) | 4785 | 3522 | 3324 |
| Incremental cost (₹ 1000s) per QALY gained (including indirect costs) | 4564 | 3266 | 3040 |

*QALY: Quality adjusted life years; ₹: Indian Rupees

**S12 Table: Summary of model predicted incremental cost and health outcomes in a cohort of 100,000 children immunized with TCV at the age of 5^th^ year (with protective efficacy for 5 years) as compared to no vaccination in urban settings of India**

| **Incremental outcomes** | **Vaccination as compared to no vaccination** |
| --- | --- |
| Typhoid cases averted (%) | 3339 (28) |
| Typhoid deaths averted (%) | 9 (28) |
| Life years gained | 51 |
| QALY gained | 69 |
| Incremental cost in ₹ 1000s (excluding indirect costs) | 8015 |
| Incremental cost in ₹ 1000s (including indirect costs) | -15,803 |
| Incremental cost (₹) per QALY gained (excluding indirect costs) | 115,908 |
| Incremental cost (₹) per QALY gained (including indirect costs) | -228,551 |

*QALY: Quality adjusted life years; ₹: Indian Rupees

**S13 Table: Summary of model predicted incremental cost and health outcomes in the newly borne cohort of 100,000 children following vaccination as compared to no vaccination in India considering a waning rate of 10% and 20% for TCV in the age group of 5-10 years and 10-15 years respectively in urban settings**

| **Incremental outcomes** | **Vaccination strategies (as compared to no vaccination)** | | |
| --- | --- | --- | --- |
|  | **Scenario 1: Protective efficacy of 5 years** | **Scenario 2: Protective efficacy of 10 years** | **Scenario 3: Protective efficacy of 15 years** |
| Typhoid cases averted (%) | 2057 (17) | 5149 (43) | 7220 (60) |
| Typhoid deaths averted (%) | 5.7 (17) | 14 (43) | 20 (60) |
| Life years gained | 54 | 103 | 112 |
| QALY gained | 67 | 132 | 152 |
| Incremental cost (excluding indirect costs) | 10,138 | 2935 | -1387 |
| Incremental cost (including indirect costs) | -4540 | -34,054 | -52,699 |
| Incremental cost (₹) per QALY gained (excluding indirect costs) | 151,346 | 22,124 | -9113 |
| Incremental cost (₹) per QALY gained (including indirect costs) | -69,293 | -256,718 | -346,386 |

*QALY: Quality adjusted life years; ₹: Indian Rupees

**S14 Table: Summary of model predicted incremental cost and health outcomes in the newly borne cohort of 100,000 children following vaccination as compared to no vaccination in India considering a waning rate of 75% in the age group of 5-10 years and zero efficacy rate thereafter in urban settings**

| **Incremental outcomes** | **Vaccination strategies (as compared to no vaccination)** | |
| --- | --- | --- |
|  | **Scenario 1: Protective efficacy of 5 years** | **Scenario 2: Protective efficacy of 10 years** |
| Typhoid cases averted (%) | 2057 (17) | 2978(25) |
| Typhoid deaths averted (%) | 5.7 (17) | 8(25) |
| Life years gained | 54 | 69.83 |
| QALY gained | 67 | 87.70 |
| Incremental cost (excluding indirect costs) | 10,138 | 8191 |
| Incremental cost (including indirect costs) | -4540 | -13314 |
| Incremental cost (₹) per QALY gained (excluding indirect costs) | 151,346 | 93,401 |
| Incremental cost (₹) per QALY gained (including indirect costs) | -69,293 | -151,808 |

*QALY: Quality adjusted life years; ₹: Indian Rupees

**S15 Table: Model predicted incremental cost effectiveness ratio by increasing the utility values of each of the health state by 6% (absolute increase) in the newly borne cohort of 100,000 children following vaccination as compared to no vaccination in India**

| **Incremental outcomes** | **Vaccination strategies (as compared to no vaccination)** | | |
| --- | --- | --- | --- |
|  | **Scenario 1: Protective efficacy of 5 years** | **Scenario 2: Protective efficacy of 10 years** | **Scenario 3: Protective efficacy of 15 years** |
| ***Panel A: Urban settings*** | | | |
| Incremental cost (₹) per QALY gained (excluding indirect costs) | 165,400 | 68,333 | 51,106 |
| Incremental cost (₹) per QALY gained (including indirect costs) | -85,788 | -234,345 | -285,450 |
| ***Panel B: Rural settings*** | | | |
| Incremental cost (₹ 1000s) per QALY gained (excluding indirect costs) | 4127 | 3066 | 2921 |
| Incremental cost (₹ 1000s) per QALY gained (including indirect costs) | 3881 | 2779 | 2598 |

*QALY: Quality adjusted life years; ₹: Indian Rupees
